# Extension of the Lecture Term

**Published:** 1848-08

**Authors:** 


### EXTENSION OF THE LECTURE TERM.

A good deal of misapprehension seems to prevail as to the action of the American Medical Association on this subject, at the meeting held in Baltimore in May last. The June number of the Western Journal of Medicine and Surgery, in a report of the proceedings of the Associ ation, contains the following remarks on the subject:—

"Dr. Wellford, of Fredericksburg, Va., now proceeded to read th report of the committee on Medical Education, of which Dr. Stevens was chairman. The report was accompanied by a number of resolutions, three of which had an important bearing on Medical Schools, and in substance are as follows: 1st.—That the plan of education in any Medical School must be considered radically defective, which does not embrace anatomical dissection by the pupils, and clinical instruction. 2d.—*That it be recommended to Medical Schools in the country which have not already done so, to extend their sessions to five months.* 3d.—That it be recommended to the Faculties of the several Medical Schools, to associate with them, in the examination of their students for the doctorate, a number of physicians not connected with the schools.

"The first of these resolutions passed unanimously. The second was adopted with little division, although it is known that not a few gentlemen in the Association, connected with Medical Schools, question the policy of lengthening the lecture term."

The Buffalo Journal for June 1848, contains the following, on the same subject:—"At the last meeting of the Association, as we learn, this subject (lengthening the term) was reconsidered, and it was concluded that, for the present, it was advisable to recommend an extension to five months only."

Although present at the meeting when the report was made we took no notes, but we were strongly impressed with the same view of what occurred as our contemporaries, and accordingly, having occasion to allude to the subject in our last number, we mentioned that the report of the committee on medical education recommended *five* months, which was concurred in by the Association. We have ascertained, however, that we were in error. We learn from Dr. Stillé, Secretary of the Association, that *five* months is not recommended either in the report of the committee or the resolutions passed by the Association, so that there has been an entire misconception on the subject. The fourth resolution, submitted by the committee and adopted by the Association, reads as follows:—"*Resolved*, That this committtee reiterate and strongly recommend to the Association, a practical observance of the Resolutions appended to the Reports of the Committees on Preliminary Education, and on the requisites for gradution, submitted to the Medical Convention which assembled in Philadelphia in May 1847."

Desirous at all times of being right, we take the earliest opportunity of correcting our own mistake, as well as the misapprehension of our brother journalists on this point.

